# Correction: The molecular identity of the TLQP-21 peptide receptor

**DOI:** 10.1007/s00018-023-04718-7

**Published:** 2023-02-20

**Authors:** Bhavani S. Sahu, Megin E. Nguyen, Pedro Rodriguez, Jean Pierre Pallais, Vinayak Ghosh, Maria Razzoli, Yuk Y. Sham, Stephen R. Salton, Alessandro Bartolomucci

**Affiliations:** 1 National Brain Research Centre, NH-8, Manesar, Gurugram, Haryana 122052 India; 2 Department of Integrative Biology and Physiology, University of Minnesota, 2231 6th St. SE, Minneapolis, MN 55455 USA; 3 Bioinformatics and Computational Biology Program, University of Minnesota, Minneapolis, USA; 4 Departments of Neuroscience and Geriatrics and Palliative Medicine, Friedman Brain Institute, Icahn School of Medicine at Mount Sinai, One Gustave L. Levy Place, New York, NY 10029 USA

**Correction to: Cellular and Molecular Life Sciences (2021) 78:7133–7144** 10.1007/s00018-021-03944-1

In this article the legend for Fig. 2 was inadvertently processed incorrectly; the Fig. 2 caption should appeared as shown below.

**Figure 2** Structure and sequence of C3aR1, gC1qR and HSPA8. **A** Left to right: 3-dimensional structures of human C3aR1 (NP_001313406.1, homology model [4]), C1qR (PDB: 6SZW [81]), and structure binding domain of HPSA8 (PDB: 4PO2 [80]). Structures are shown as ribbon representation and colored by chain. **B** Multiple sequence alignment of human and mouse sequences of C3aR1, C1qR, and HSPA8. Alignment performed with MUSCLE in Schrödinger Multiple Sequence Viewer (Maestro, Schrödinger, LLC, New York, NY, 2020) with 10.0 opening gap and 0.20 extending gap penalties and manually inspected. Only sequence regions within 10 residues of C3aR1 key binding site residues are shown for simplicity. Grey rectangles indicate extension of sequences not shown. Black outline indicates C3aR1 residues that form salt bridge interactions with TLQP-21 pharmacophore

